# Real-life experience with 4 years of golimumab persistence in ulcerative colitis patients

**DOI:** 10.1038/s41598-020-73577-0

**Published:** 2020-10-20

**Authors:** Marisa Iborra, Natalia García-Morales, Saoia Rubio, Federico Bertoletti, Marta Calvo, Carlos Taxonera, Marta Maia Boscá-Watts, Mónica Sierra, Noemí Mancenido, Belén Beltrán, Óscar Nantes Castillejo, Esther García-Planella, Isabel Vera, Cristina Alba, David Martí-Aguado, María Pilar Ballester, Noelia Cano-Sanz, Ramón Pajares-Villarroya, Elena Cerrillo, Antonio Cañada, Pilar Nos

**Affiliations:** 1 Department of Gastroenterology, Hospital Universitario y Politécnico La Fe, Avinguda de Fernando Abril Martorell, 106, 46026 Valencia, Spain; 2 Centro de Investigación Biomédica en Red de Enfermedades Hepáticas y Digestivas (CIBERehd), Madrid, Spain; 3 Department of Digestive Diseases, Complejo Hospitalario de Navarra, Pamplona, Spain; 4 Department of Gastroenterology, Hospital de la Santa Creu i Sant Pau, Barcelona, Spain; 5 Department of Gastroenterology, Hospital Universitario Puerta de Hierro, Majadahonda, Madrid, Spain; 6 Department of Gastroenterology, Hospital Clínico San Carlos, Madrid, Spain; 7 Department of Gastroenterology, Hospital Clínico Universitario, Valencia, Spain; 8 Department of Gastroenterology, Complejo Asistencial Universitario de León, León, Spain; 9 Department of Gastroenterology, Hospital Universitario Infanta Sofía, San Sebastián de los Reyes, Madrid, Spain; 10 Deparment of Biostatistics and Bioinformatics, Instituto de Investigación Sanitaria Hospital Universitario y Politécnico La Fe, Valencia, Spain

**Keywords:** Inflammatory bowel disease, Crohn's disease, Ulcerative colitis

## Abstract

Golimumab has demonstrated its long-term efficacy and safety in ulcerative colitis in clinical trials, but no data of long-term persistence has been published from real world. To estimate long-term persistence of golimumab, as well as factors associated with longer persistence, in patients with ulcerative colitis in real life. Observational multicentre study including adult patients with ulcerative colitis treated with golimumab and with at least twelve months of follow-up. We included 190 patients, 105 (55.26%) naive to anti-TNF, with mean disease duration of 9.32 ± 8.09 years. Probability of persistence was 63%, 46%, 39% and 27% at 1, 2, 3 and 4 years, respectively. Persistence was lower in patients with primary failure to previous anti-TNF. Eighty-two (43.16%) patients needed dose intensification during follow-up, with a mean time until intensification of 8.03 ± 8.64 months. Dose intensification and lower disease duration predicted higher persistence with golimumab (p = 0.037 and p = 0.008, respectively). During a follow-up of 17.25 ± 15.83 months, 32 (16.5%) patients needed hospitalisation and 11 (6%) underwent colectomy. No unexpected adverse events were reported. Golimumab has demonstrated good persistence and safety profile for long treatment in ulcerative colitis patients.

## Introduction

Golimumab has demonstrated its efficacy and safety in moderately to severely active ulcerative colitis (UC) in clinical trials (PURSUIT, Programme of Ulcerative Colitis Research Studies Utilising an Investigational Treatment), both at inducing and maintaining clinical response^1,2^. Also, in the long term extension study, 63% of patients remained on golimumab treatment after more than 4 years of therapy^3^. However, patients included in clinical trials do not represent real UC patients. Thus, it is important to know real-life efficacy and safety of treatments.

Persistence is defined as the time from the beginning to the discontinuation of a treatment^4^. It is related to efficacy, safety and adherence of therapy^4,5^. In a retrospective study, persistence rates of UC patients were lower than those in Crohn’s disease (CD) patients^5^. Probability of persistence with golimumab was similar to other anti-TNFs in UC patients, with persistence at 1 year around 40–45% for infliximab, adalimumab and golimumab. Hospitalisations, corticosteroid use and infections (septicaemia, *Clostridium difficile* infection and pneumonia) were the risk factors for non-persistence, whereas early initiation of immunosuppressants and dose escalation were protective factors^5^. However, there are no real-world data on the persistence of golimumab in UC patients beyond 2 years^5–9^.

Therefore, the primary aim of this study was to estimate the probability of long-term persistence with golimumab therapy in patients with UC in real life. Secondary aims were to ascertain rates of hospitalisation, surgery, and adverse events during follow-up; to establish if there are differences between patients previously treated with anti-TNF and naive patients; and to assess possible factors associated with long-term persistence of golimumab.

## Materials and methods

### Characteristics of study population

Inclusion criteria were all patients aged 18 years or older diagnosed with UC according to standard criteria^10^, that received golimumab treatment based on the decision of the attending physician in daily clinical practice and the drug SPC (summary of product characteristics) with at least 12 months of follow-up. We excluded patients who were under 18 years old, without confirmed UC diagnosis, or patients in which the main indication of golimumab was other than UC (i.e. other concomitant immune-mediated disease).

We recorded demographic and clinical data such as gender, age at UC diagnosis, UC duration and disease extent according to the Montreal classification^11^. We also recorded previous and concomitant treatments at baseline, and reason for withdrawal in patients previously exposed to another anti-TNF. Adverse events, hospitalisations, and colectomies during follow up were also recorded during follow-up.

### Definitions^12^

Primary non-response was defined as the absence of clinical and biochemical improvement and subsequent discontinuation of the drug during induction therapy.

Secondary non-response was defined as discontinuation of the drug due to secondary loss of response after improvement during induction.

Intolerance was defined as discontinuation of the treatment owing to AEs.

When it was possible, therapeutic drug monitoring (TDM) was used to better define the causes and management of the primary non-response or secondary loss of response to previous anti-TNF (infliximab, adalimumab).

Golimumab treatment was discontinued based on the discretion of the treating physician as golimumab are not available in routine clinical practice. Furthermore, currently, the ranges of golimumab through levels are not clearly defined.

### Study design

We designed an observational, multicentre cohort study with retrospective data collection of patients with UC, treated with golimumab in inflammatory bowel disease (IBD) units, from eight Spanish hospitals. We included data since golimumab approval (May 2014) until October 2018. Data were collected from clinical charts from every participant hospital.

### Statistical analysis

We recorded data in Excel sheets. We expressed the results as mean and standard deviation (SD) and median and interquartile range (IQR) for continuous variables, and as absolute and relative frequencies for categorical variables. A p < 0.05 was considered statistically significant.

To estimate the risk of discontinuing golimumab, we performed a multivariable analysis (Cox regression) adjusted for confounding factors (use of previous anti-TNF agents, concomitant immunosuppressive treatment at baseline, dose intensification, and disease extent and duration). Additionally, we performed a Cox model to assess differences in risk of discontinuing treatment according to the previous exposure to anti-TNF and the cause of withdrawal of previous anti-TNFs. Kaplan–Meier curves were built. The differences between the population survival curves were performed by log-rank test. A p-value of less than 0.05 was considered statistically significant.

All statistical analyses were performed using R (version 3.5.1) and R package ordinal (version 2018.4-19)^13^.

### Ethical statement

The Ethics Committees of all participating hospitals (Hospital Universitario y Politécnico La Fe, Valencia; Complejo Hospitalario de Navarra, Pamplona; Hospital de la Santa Creu i Sant Pau, Barcelona; Hospital Universitario Puerta de Hierro de Madrid; Hospital Clínico San Carlos, Madrid; Hospital Clínico Universitario de Valencia; Complejo Asistencial Universitario de León; and Hospital Universitario Infanta Sofía, San Sebastián de los Reyes) reviewed and approved the study protocol. All included patients signed an informed consent authorizing the use of their clinical data for research purposes. Additionally, the study complies with the principles of Good Clinical Practice and the Helsinki Declaration.

## Results

### Patient characteristics

We included 190 patients with UC; 53% were male, the mean age was 44.39 ± 15.33 years and the age at UC diagnosis was 35.2 ± 14.23 years. Patients had mean UC duration of more than 9 years and extensive UC extent in almost 60% of cases.

More than 10% of patients had some concomitant immune-mediated disease: rheumatoid arthritis (7 patients, 3.7%), psoriasis (5 patients, 2.6%), ankylosing spondylitis (3 patients, 1.6%), and other diseases (5 patients, 2.6%).

As regards to the number of previous anti-TNFs, 105 (55%) patients were naive to anti-TNFs, 50 (26%) patients had received one, 33 (18%) patients had been treated with two, and 2 (1%) patients had received three previous anti-TNFs. No patient had previously been treated with vedolizumab. Discontinuation of previous anti-TNFs in exposed patients (n = 85) was due to primary failure in 25 (29%) patients, loss of response (secondary failure) in 43 (51%) patients, adverse events in 12 (14%) patients, and preference for subcutaneous administration in 5 (6%) patients. The demographic and clinical characteristics of the anti-TNF experienced and naive patients were similar (Table 1).

**Table 1:** Demographic and clinical characteristics of study population.

	Global (n = 190)	Anti-TNF naïve (n = 105)	Previously treated with an anti-TNF (n = 85)
Male/female	101 (53%)/89 (47%)	58 (55%)/47 (45%)	43 (51%)/42 (49%)
Age, years	44.39 ± 15.33 43.26 (33.61, 54.89)	44.02 ± 16.69 42.9 (33.08, 54.9)	44.85 ± 13.53 43.57 (34.32, 53.9)
Disease duration, years	9.32 ± 8.09 7.52 (2.99, 12.78)	8.52 ± 8.19 6.25 (1.97, 2.79)	10.31 ± 7.89 8.36 (4.79, 12.75)
**Disease extent (montreal classification)**			
E1 (proctitis)	7 (4%)	4 (4%)	3 (4%)
E2 (left-side colitis)	74 (39%)	47 (45%)	27 (32)
E3 (extensive colitis)	109 (57%)	54 (51%)	55 (64%)
Concomitant autoimmune disease	20 (10.5%)	98 (93%)	72 (85%)
Concomitant immunosuppressants	91 (48%)	54 (51%)	37 (44%)
Concomitant corticosteroids	72 (39%)	37 (37%)	35 (42%)
**Indication for golimumab**			
Induction of remission	172 (90%)	102 (97%)	70 (82%)
Maintenance of remission	16 (9%)	2 (2%)	14 (17%)
Extraintestinal manifestations	2 (1%)	1 (1%)	1 (1%)
**Induction dose**			
200/100 mg	184 (98%)	103 (100%)	81 (96.4%)
**Maintenance dose**			
100 mg every 4 weeks	87 (47%)	50 (49%)	37 (44%)
50 mg every 4 weeks	99 (53%)	52 (51%)	47 (56%)

Data are expressed as mean ± standard deviation and median (interquartile range), or absolute number (%).

At baseline, nearly 50% of patients received immunosuppressants: azathioprine in 73 (80.2%) patients, mercaptopurine in 11 (12.1%) patients, methotrexate in 5 (5.5%) patients, and other drugs in 2 (2.2%) patients. Moreover, almost 40% of patients were treated with corticosteroids at the beginning of golimumab treatment. See detailed data in Table 1.

The main indication for golimumab therapy was induction of remission in more than 90% of patients, all of them with partial Mayo Score > 2. Golimumab was administered to 16 patients as maintenance of remission (partial Mayo Score ≤ 2 with no individual subscore > 1), which means that previous anti-TNF therapies achieved the clinical remission but were withdrawn due to other causes mainly adverse events, poor venous access or preference for subcutaneous administration. Almost all patients received the usual dose for induction (200–100 at weeks 0 and 2). As maintenance dose, one half of the patients received 50 mg every 4 weeks and the other half received 100 mg every 4 weeks. Eighty-two (43.0%) patients needed dose escalation from 50 to 100 mg every 4 weeks during follow-up, with a mean time until intensification of 8.03 ± 8.64 months. Finally, golimumab maintenance therapy had a mean duration of almost 18 months. Golimumab was discontinued in almost 60% of patients during the follow-up. Main reason for discontinuation was primary failure, especially among biologic-naive patients (Table 2).

**Table 2:** Management and causes of treatment discontinuation during follow-up.

	Global (n = 190)	Anti-TNF naïve (n = 105)	Previously treated with an anti-TNF (n = 85)
Intensification	82 (43%)	45 (43%)	37 (44%)
Time until intensification, months	8.03 ± 8.64 4.6 (2.37, 10.1)	7.67 ± 9.46 3.94 (2.3, 7.29)	8.46 ± 7.67 5.04 (3.07, 10.44)
Duration of golimumab maintenance, months	17.25 ± 15.83 12.04 (4.39, 26.28)	15.68 ± 14.28 9.95 (4.01, 22.54)	19.3 ± 17.47 13.01 (4.99, 34.1)
Discontinuation of golimumab	107 (56%)	57 (54%)	50 (59%)
**Cause of discontinuation of golimumab**			
Primary failure	62 (58%)	40 (70%)	22 (44%)
Loss of response	34 (32%)	11 (19%)	23 (46%)
Adverse events	11 (10%)	6 (11%)	5 (10%)

Data are expressed as mean ± standard deviation and median (interquartile range), or absolute number (%).

### Probability of persistence with golimumab and risk factors

**Figure 1:**
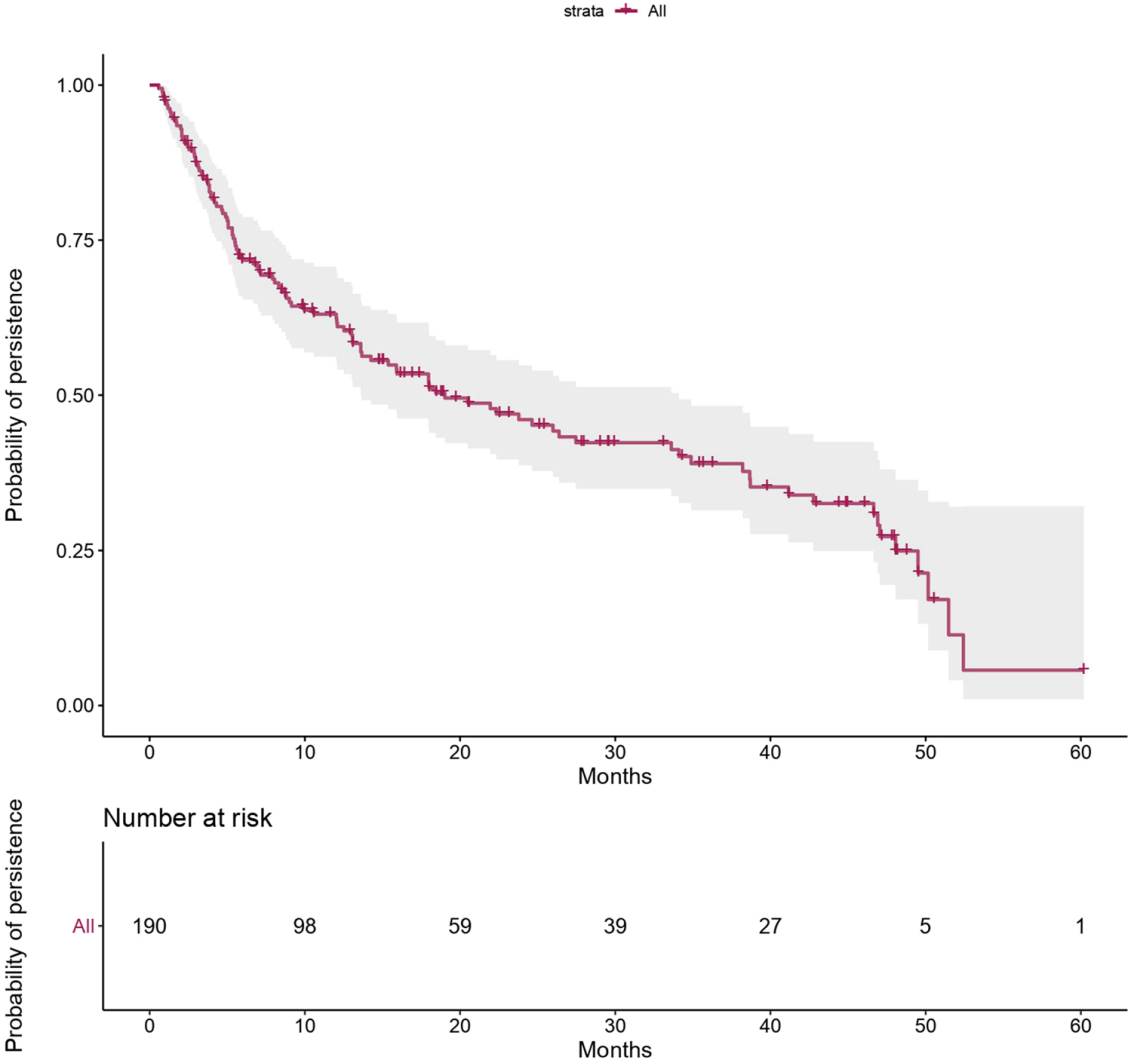
Probability of persistence with golimumab in long-term maintenance treatment of ulcerative colitis. The figure shows a Kaplan–Meier curve of probability of persistence for all patients during follow-up. Statistical analysis was performed using R Software^13^.

**Figure 2:**
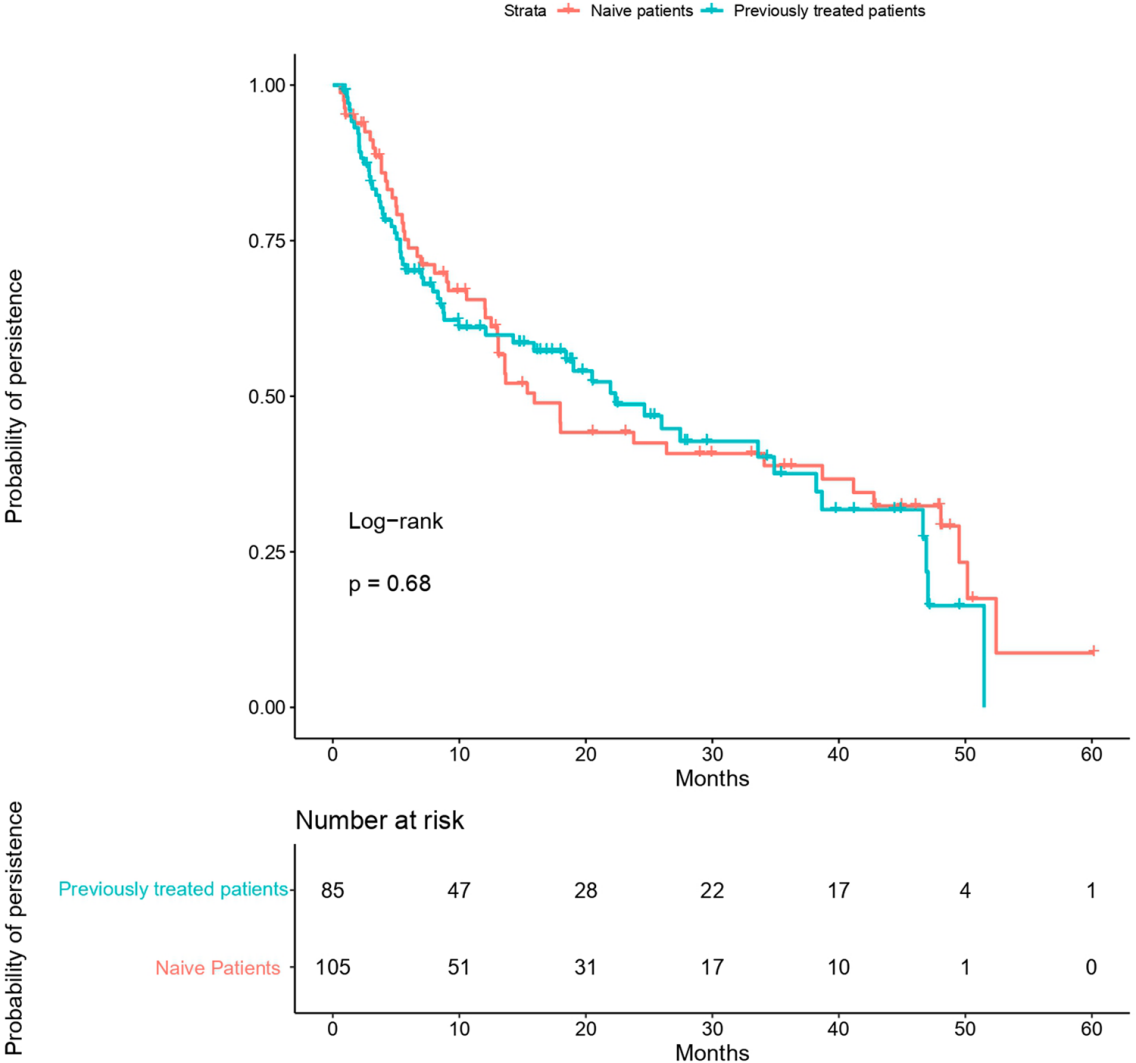
Probability of persistence in naive and previously treated patients. The probability of persistence was similar to previously anti-TNF treated patients and anti-TNF naive patients (p = 0.68 by log-rank test). Statistical analysis was performed using R Software^13^.

**Figure 3:**
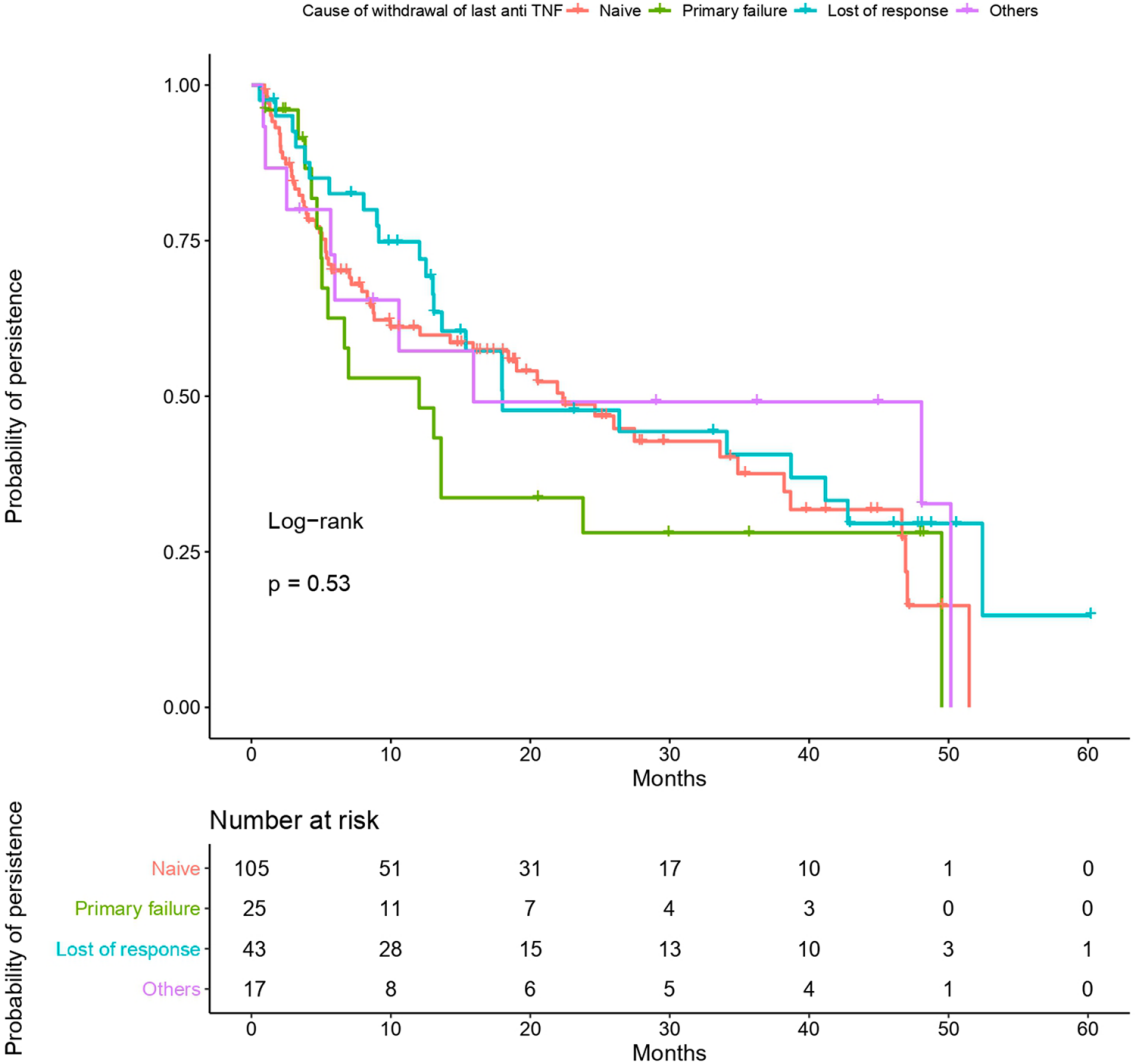
Probability of persistence according to the cause of withdrawal of last anti-TNF. The persistence did not show statistical differences among patients who stopped prior anti-TNF by primary failure, secondary failure or other causes (p = 0.53 by log-rank test). Statistical analysis was performed using R Software^13^.

Probability of persistence with golimumab, assessed with an adjusted Cox model, was 63% at 1 year, 46% at 2 years, 39% at 3 years and 27% at 4 years. Figure 1 shows Kaplan–Meier curve of probability of persistence for all patients. Moreover, probability of persistence was similar in previously anti-TNF treated patients and anti-TNF naive patients (p = 0.68) (Fig. 2). No differences were found according to the number of prior anti-TNFs in previously treated patients. However, persistence was lower in patients who had primary failure to prior anti-TNF but no significantly differences were found (p = 0.53) (Fig. 3).

Multivariate analysis showed that dose intensification and lower disease duration were associated with a higher golimumab persistence (p = 0.037 and p = 0.008, respectively) (Table 3). Prior use of anti-TNFs, concomitant immunosuppressant use, and disease extent were not associated with higher persistence.

**Table 3:** Cox model to assess factors associated with higher persistence with golimumab treatment.

Variables	HR	Lower 95% CI	Upper 95% CI	P
Previous biological agents	1.094	0.742	1.613	0.651
Concomitant immunosuppressants	1.183	0.806	1.738	0.391
Dose intensification	1.519	1.025	2.249	**0.037**
Disease extent: left-side colitis	1.556	0.556	4.351	0.40
Disease extent: extensive colitis	1.486	0.536	4.116	0.446
Disease duration	0.961	0.933	0.99	**0.008**

Statistically significant results are given in bold.

*HR* hazard ratio, *CI* confidence interval.

### Safety

There were 34 reported adverse events in 31 patients during follow-up (Table 4). Although adverse events used to be mild, none of them required patient hospitalisation and 10 forced golimumab discontinuation. There were only two severe infections and both required hospitalisation and golimumab discontinuation. No malignancies or deaths were reported.

**Table 4:** Reported adverse events with long-term golimumab treatment.

Type of adverse event	N (%)
**Infections**	
Respiratory: mild respiratory infections (3), tracheobronchitis (1), pneumonia (1^a,b^), pneumonitis (1), influenza (1)	15 (44.1%)
Urinary: urinary tract infections (5)	
Gastrointestinal: acute gastroenteritis (1)	
Otolaryngology (ENT): tonsillitis (1)	
Others: mononucleosis (1^b^)	
**Skin process**	
Rash at the injection site (1), folliculitis (1), pruritus and urticaria (1), eczema-like rash with psoriatic distribution (2^a^), rash (1)	6 (17.6%)
**Neurological processes**	
Myelitis (2^a,b^), headaches (2), dizziness (1)	5 (14.7%)
**Arthropathies**	
Reactive knee arthritis (1^a,b^), arthralgia (1)	2 (5.8%)
**Heart disease**	
Heart failure (1^a,b^)	1 (2.9%)
**Others**	
Lupus-like condition (1^a,b^), leukopenia (1), fever (2^a,b^), bronchospasm (1)	5 (14.7%)
Total	34 (100%)

Data are expressed as absolute number in each adverse event (n) and absolute number (%) in each type of adverse event.

^a^Adverse event that caused golimumab discontinuation.

^b^Adverse event that caused patient hospitalization.

During follow-up, 16.5% of patients needed UC-related hospitalisation and 5.8% of patients underwent surgery (colectomy). Motives of hospitalisation were adverse events in 10 patients, surgery in 11 patients and disease flare-up in other 11 patients. Naive patients had more adverse events but lower rates of UC-related hospitalisation and colectomy. See detailed data in Table 5.

**Table 5:** Rates of adverse events, UC-related hospitalisations and colectomies in the study population.

Safety	Global (n = 190)	Anti-TNF naïve (n = 105)	Previously treated with an anti-TNF (n = 85)	p
Adverse events	31 (17.1%)	21 (21.4%)	10 (12%)	0.12
Hospitalizations	32 (16.8%)	15 (14.3%)	17 (20%)	0.29
Surgery	11 (5.8%)	4 (3.8%)	7 (8.2%)	0.18

## Discussion

We assessed the persistence with golimumab up to 4 years in patients with UC in real world. We also analysed factors related to persistence. To our knowledge, this is the first study of golimumab in UC with such a long follow-up. Our results showed that persistence ranged from 63% at 1 year to 27% at 4 years, without differences between anti-TNF naive and experienced patients. The lower persistence was found in patients with primary failure to previous anti-TNF. As predictive factors of longer persistence, we found dose intensification and lower disease duration.

In the long-term extension of PURSUIT-M trial, 63% of patients remained on golimumab treatment after 228 weeks of therapy^3^. No real-world studies have been focused on golimumab persistence beyond 1–2 years. In our population, persistence at 1 year (63%) was higher than in other real-life studies. In the SMART study (n = 91), only 32% of patients were on golimumab at week 52^14^. In another study, persistence was approximately 40%^5^. However, our results are similar to the study published by Bressler et al. over a national case management programme that described a cumulative probability of 63% to remain on therapy at 1 year^7^. Similarly, Taxonera et al. described that the 72% of UC patients maintaining clinical response at 1-year follow-up^8^. In contrast, persistence with golimumab has extensively been assessed in patients with rheumatic diseases; studies have demonstrated higher rates of persistence than in inflammatory bowel diseases: 85.9% at 1 year, around 70% at 2 years, 62% at 3 years and 57% at 5 years^15–17^.

In our study, golimumab was used in 55% of biological-naive patients and the probability of persistence was similar to anti-TNF experienced patients. In this sense, it has been observed that the persistence is greater in naive patients at the beginning of anti-TNF treatment. However, at long-term (2 years) biological-naive patients have similar percentages of discontinuation than anti-TNF previously exposed patients^5^. An Italian study, that included 59 UC patients that received golimumab with a mean treatment duration of 52 weeks, neither found differences between naive and experienced patients^18^. This finding has been verified recently^19^. Data of rheumatic diseases are also controversial. Although some studies have demonstrated higher persistence of golimumab in biologic-naïve patients compared with biologic-experienced patients, other studies did not find statistically significant differences between both groups of patients^15,17–22^.

We found two factors associated with higher persistence with golimumab: dose intensification and shorter disease duration. Similarly, Chen et al. showed that dose escalation of biologics increased the probability of persistence by 45% over the following 30 days^5^. In our study, 43% of patients received dose intensification during follow-up. A recent study has demonstrated that early dose intensification was effective in non-responders to induction treatment with golimumab^23^. On the other hand, our group published that early dose optimization before week 14 induced long-term clinical benefit in 54% of patients with UC^8^. Therefore, we should always consider dose intensification before golimumab discontinuation, because a high percentage of patients would benefit both short-term efficacy and long-term persistence.

Our study did not show that concomitant immunosuppressive therapy increased golimumab persistence. By contrast, in the cited systematic review, risk of discontinuation was significantly lower in patients receiving combination therapy with immunosuppressants and anti-TNFs. However, the authors did not perform the analysis separating by the different anti-TNFs due to insufficient sample size in patients initiating golimumab^5^. Other studies did not assess the role of concomitant immunosuppressants^7,13^. Combination of anti-TNF agents and immunosuppressants may improve response^23,24^, but there are potential adverse events such as greater risk of infections and some malignancies^25,26^.

We also assessed safety of golimumab. No unexpected adverse events were reported. In our population, experienced patients had more adverse events, but other studies did not find differences according to previous exposure to anti-TNF agents^6^. Regarding colectomy and hospitalisation rates, both were low and similar to those in other real-world studies^27^. Other studies have also found a lower rate of surgery in naive patients^6,18^.

Strengths of our study derived from its chart review design. It allowed to collect routinely recorded information, minimised recall bias and implied less resource consumption that other types of design^28^. However, this design also entailed some weaknesses or limitations, such as that extraction and interpretation of data could vary, and that some charts could be incomplete or even lost^28^. Another potential limitation of our study was that golimumab discontinuation was at the discretion of participant physicians. This may explain the high percentage (70%) of primary failure in non-anti-TNF experienced patients, described in our population. In addition, we did not measure anti-golimumab antibody titres or serum levels of golimumab. It has been suggested that efficacy of anti-TNF treatments may be affected by the presence of anti-drug antibody titres and low serum levels^29,30^. Therapeutic drug monitoring has achieved a central role in clinical management of biologic agents, because dose adjustments can improve outcome when there is primary failure or loss of response. Lower serum level of golimumab correlated with worse clinical and endoscopic efficacy in clinical studies^1,2^ and real world^30–33^. However, normal values have not been clearly defined and these parameters are not used in routine clinical practice. Finally, we did not assess the correlation between persistence and endoscopic remission. In the SMART study, the only predictive factor of discontinuation-free survival was short-term mucosal healing at week 14^14^.

Our real-life study showed that around 30% of patients with UC maintained golimumab at 4-year follow-up especially those with dose escalation and shorter disease duration, and without differences between previously anti-TNF exposed and naive patients. Thus, we conclude that golimumab has demonstrated good persistence in UC. Factors related to persistence of golimumab in these patients merit further investigation, as well as the role of serum golimumab levels in order to improve management and increase persistence.
